# Improving Semen Quality in a Male Partner With Abnormal Seminal Parameters Through Yoga and Yoga Nidra: A Case Report

**DOI:** 10.7759/cureus.54095

**Published:** 2024-02-12

**Authors:** Ritesh Jadhav, Akash More, Shilpa Dutta, Gauri Gajabe, Jarul Shrivastava, Saurabh Mehakar

**Affiliations:** 1 Clinical Embryology, Datta Meghe Institute of Higher Education and Research, Wardha, IND

**Keywords:** yoga nidra, male factor infertility, lifestyle choices, psychological health, complementary therapies, yoga

## Abstract

This research presents a case study involving a 39-year-old male and his 34-year-old female partner seeking fertility consultation in Maharashtra, India, after struggling to conceive for over three years. Despite the male participant's lack of discernible medical conditions and typical lifestyle, semen analysis revealed severe oligozoospermia attributed to elevated stress levels from his physically demanding occupation and infertility-related emotional strain. The female partner exhibited normal blood parameters, including anti-Müllerian hormone (AMH). Embracing holistic approaches, the couple integrated yoga and Yoga Nidra into their daily routine to address stress-induced hormonal imbalances. The customized yoga regimen is aimed at stress reduction and overall well-being, incorporating physical postures, breathing exercises, and meditation. Yoga Nidra, a guided relaxation technique, was employed to induce profound rest and alleviate stress. Over a 12-week period, the male participant diligently adhered to the regimen, reporting heightened relaxation, improved sleep quality, and reduced stress levels. Semen analysis before and after intervention showed significant improvements in sperm count and motility alongside diminished morphological abnormalities.

In parallel, the female partner underwent intrauterine insemination (IUI), resulting in a positive beta-human chorionic gonadotropin (β-hCG) analysis. Weekly follow-ups monitored progress, with supplementation administered as needed. While promising, further research with larger sample sizes and controlled trials is warranted to establish definitive efficacy. Overall, yoga and Yoga Nidra offer noninvasive adjuncts to conventional therapies for male infertility, underscoring the importance of integrating holistic practices into comprehensive fertility management strategies.

## Introduction

Infertility concerns often encompass male factors, with abnormal seminal characteristics such as low sperm counts, poor morphology, and reduced motility significantly impacting conception [1]. While conventional medical treatments exist, complementary modalities like yoga and Yoga Nidra offer a holistic approach to enhancing overall health and, potentially, fertility [2]. This case study explores the effects of integrating yoga and Yoga Nidra into the daily routine of a man with aberrant seminal parameters.

Salambha Sarvangasana (supported shoulderstand), Bhujangasana (cobra pose), and Baddha Konasana (bound angle pose) were observed to augment sperm count potentially. However, scientific evidence directly linking these yoga poses to increased sperm count remains limited [3]. Yoga, renowned for its multifaceted benefits in improving overall health and well-being, including stress reduction, enhanced circulation, and hormonal balance, presents a promising avenue [2]. However, claims regarding specific poses' direct impact on reproductive health warrant cautious interpretation due to the multifactorial nature of sperm count regulation.

Male fertility is intricately influenced by genetics, lifestyle, diet, and overall health [4]. While regular physical activity, stress reduction techniques, and a balanced lifestyle contribute to better reproductive health, attributing these effects solely to specific yoga poses oversimplifies the complex interplay of factors involved. Nonetheless, alternative and complementary therapies like yoga have garnered attention for their potential to improve male reproductive health through stress reduction, hormone balance, and overall well-being [5].

Yoga Nidra, a deep relaxation technique rooted in yoga principles, offers additional psychological and physical benefits by inducing a state of conscious relaxation while maintaining awareness [2]. Their study delves into the journey of a man with abnormal seminal parameters who adopted a disciplined yoga and Yoga Nidra regimen to improve the quality of his semen [2]. The research primarily examines the impact of these practices on semen quality indicators, shedding light on potential repercussions for both physical and psychological health [6].

## Case presentation

Couple-specific information

This research entails a 39-year-old male and his 34-year-old female partner, who sought consultation at a fertility institution situated in Maharashtra, India. The couple had been endeavoring to achieve conception for a period exceeding three years. The male partner didn't have any comorbid condition, was a nondiabetic and non-smoker, and used to consume alcohol once a month. All studied parameters (luteinizing hormone [LH], follicle-stimulating hormone [FSH], progesterone levels, and transvaginal sonography on day 2 of the menstrual cycle) to diagnose the cause of infertility were normal in the female partner. The male participant displayed no discernible medical conditions, and their lifestyle was found to be consistent with typical norms. The female partner exhibited normal blood serum parameters, including FSH, LH, and AMH levels, and reported no history of surgical interventions. The male participant had a body mass index (BMI) of 23.5 kg/m^2^, while his female partner had a BMI of 24.8 kg/m^2^, indicating normal body stature.

Clinical findings

Semen analysis was conducted on the male participant, revealing indicators of compromised fertility, including a low sperm count, diminished motility, and an increased prevalence of morphological abnormalities. The male participant was diagnosed with severe oligozoospermia, characterized by a sperm count of 5 million/mL, motility of 36%, and a 90% defect rate, coupled with the presence of debris. These deviant seminal parameters observed in the male participant were attributed to his elevated stress levels stemming from occupational demands, specifically engaging in physically demanding work within coal mines, compounded by the emotional strain associated with infertility. Additionally, clinical findings underscored the female participant's anti-Müllerian hormone (AMH) concentration within the normal range, measuring 1.5 ng/mL. They were diagnosed with primary infertility.

Diagnosis and treatment

In light of the absence of discernible medical etiologies, the couple expressed receptivity toward exploring alternative interventions. During consultations, the male partner conveyed an interest in holistic approaches, prompting consideration for the integration of yoga and Yoga Nidra into their daily regimen. The rationale behind this recommendation stemmed from the potential of addressing stress-induced hormonal perturbations that might underlie their abnormal seminal parameters.

**Figure 1:**
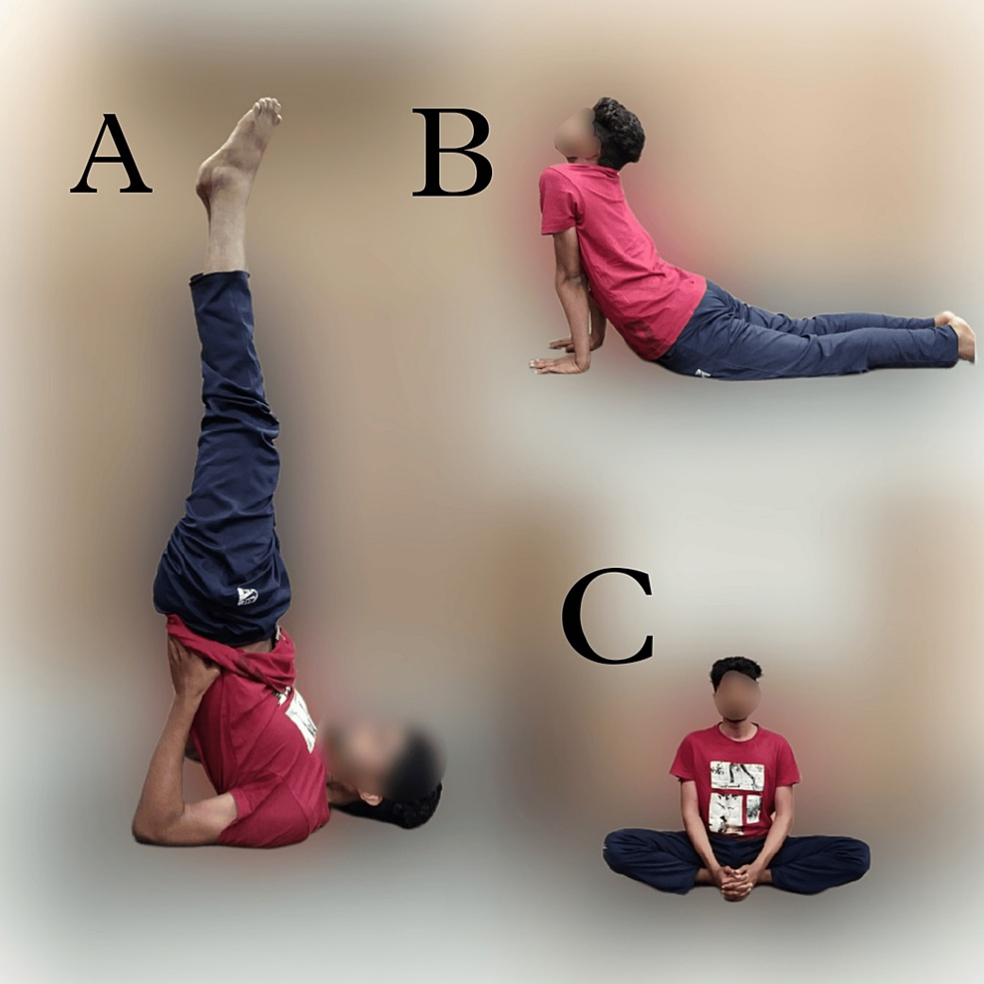
The yoga asanas performed by the male patient A) Salambha Sarvangasana, B) Bhujangasana, C) Baddha Konasana. Photos were captured by the author, and a written informed consent was obtained for publication from the patient.

The male partner participated in a customized yoga regimen one hour per day tailored to mitigate stress and promote overall well-being. This regimen encompassed yoga asanas (physical postures), pranayama (breathing exercises), and dhyana (meditation). Specifically, the inclusion of Salambha Sarvangasana, Bhujangasana, and Baddha Konasana proved beneficial in augmenting sperm count [3]. The patient performed prescribed yoga, as shown in Figures 1A-1C. While regular physical activity, stress mitigation strategies, and healthy lifestyle practices can contribute to enhanced reproductive health, attributing causality exclusively to specific yoga postures may oversimplify the multifactorial nature of this process. Poses emphasizing the pelvic region were strategically incorporated into the yoga practice to stimulate blood circulation to the reproductive organs. 

Concurrently, regular sessions of Yoga Nidra were introduced to alleviate stress and induce relaxation. Yoga Nidra, a guided relaxation technique, aims to induce a state of consciousness between wakefulness and sleep, facilitating profound rest and stress alleviation. This intervention spanned a duration of 12 weeks, during which the male participant diligently adhered to the prescribed yoga routine. Positive outcomes were reported, including heightened relaxation, improved sleep quality, and diminished daily stress levels. Notably, motility exhibited significant enhancement, with a notable increase in the proportion of actively mobile sperm. Concurrently, the frequency of morphological abnormalities diminished, approximating reference parameters.

Furthermore, the couple noted a reduction in overall tension and an enhancement in emotional connectedness. The male participant engaged in a structured yoga regimen comprising asanas and pranayama exercises aimed at optimizing circulation and stress reduction. Alongside, regular sessions of Yoga Nidra were incorporated, a guided relaxation technique recognized for its stress-alleviating properties. These interventions were implemented a minimum of five times weekly under the supervision of a yoga teacher over a 12 weeks duration. Seminal analysis was conducted both at the outset and conclusion of the intervention period, with the couple reporting augmented overall well-being and reduced stress levels. Semen analysis was performed as per the WHO 2020 guidelines. Table 1 represents the semen analysis report details taken before and after the intervention.

**Table 1:** Sperm count and motility before and after the intervention (yoga and Yoga Nidra) This table represents before and after semen analysis report details of the patient.

Before intervention		Normal range
Semen volume	1.3 milliliter	1.5-2.0 milliliter
Sperm count	5 millions/milliliter	15 million/milliliter or more
Vitality	48%	58% or more
Progressive motility	24%	32% or more
Morphological defects	89%	96% or less
pH	7.3	7.2-7.8
Post-intervention		
Semen volume	1.5 milliliter	1.5-2.0 milliliter
Sperm count	13 millions/milliliter	15 million/milliliter or more
Vitality	60%	58% or more
Progressive motility	38%	32% or more
Morphological defects	86%	96% or less
pH	7.2	7.2-7.8

The hormonal profile was normal except for cortisol, which is also considered a stress hormone. All values of diagnosed hormones are mentioned in Table 2. To reduce confounding factors, we performed a saliva cortisol test in the early morning before and after the intervention.

**Table 2:** Hormone profile of the male partner Nanomoles per liter (nmol/L), nanograms per milliliter (ng/ml)

Before intervention	
Saliva cortisol level	10.1 ng/ml
Testosterone	18 nmol/l
Follicle-stimulating hormone	1.3
Post-intervention	
Saliva-cortisol level	6.3 ng/ml
Testosterone	18 nmol/l
Follicle-stimulating hormone	1.2

Follow-up and outcome

The female participant underwent intrauterine insemination (IUI). Post-procedure assessment occurred at a 14-day interval to ascertain adherence to the prescribed medication regimen. Blood specimens were obtained for β-human chorionic gonadotropin (β-HCG) analysis, which came out to be positive. Subsequently, participants received supplementation comprising iron, vitamins, and minerals via injections and oral administration. Weekly follow-up appointments were recommended for ongoing monitoring.

## Discussion

The observed enhancement in semen quality following the integration of yoga and Yoga Nidra suggests a potential correlation between these practices and male reproductive health [7]. Yoga likely promotes sperm production and functionality by augmenting blood circulation to the reproductive organs. Moreover, given the detrimental impact of chronic stress on fertility, stress mitigation techniques like Yoga Nidra may ameliorate sperm parameters [8]. Specific yoga asanas such as Salambha Sarvangasana, Bhujangasana, and Baddha Konasana have demonstrated efficacy in increasing sperm count. Generally, yoga confers manifold physical and mental health benefits. Consistent yoga practice may foster overall well-being and stress reduction, indirectly influencing reproductive health. Stress alleviation, in particular, could play a pivotal role in supporting reproductive function [9].

Recent attention has focused on the potential influence of alternative activities like yoga and Yoga Nidra on male reproductive health [9]. Their potential effects on enhancing semen quality in males exhibiting abnormal seminal parameters are explored in the case discussion. Semen quality is paramount for reproductive success, and deviations from the norm may impede conception. Environmental factors, stress, and lifestyle choices can all impact semen quality. Yoga, encompassing physical postures, breathing exercises, and meditation, has been proposed as a means to mitigate these effects [10]. Certain yoga poses and pranayama techniques may augment blood flow to the reproductive organs and mitigate oxidative stress, potentially enhancing semen properties. Additionally, stress reduction through yoga may positively influence hormonal balance, indirectly benefiting semen quality [11].

Yoga Nidra, a guided form of meditation, may enhance semen characteristics and restore hormonal equilibrium by reducing stress. However, empirical evidence regarding the direct impact of yoga and Yoga Nidra on semen quality remains scarce [12]. While some studies have reported associations between regular yoga practice and improved semen parameters, inconsistencies in sample sizes and methodologies warrant caution. Individual responses to these interventions may vary [13]. In cases of severe sperm abnormalities, medical interventions such as assisted reproductive techniques may be necessary. Therefore, yoga and Yoga Nidra should be considered adjunctive rather than standalone therapies. Overall, yoga and Yoga Nidra show promise as complementary approaches to enhancing semen quality in males with abnormal seminal parameters [12]. They may promote reproductive health by mitigating stress, improving blood circulation, and restoring hormonal balance. However, comprehensive studies, including randomized controlled trials with larger sample sizes, are needed to ascertain their efficacy [14]. Couples seeking to enhance fertility should consult medical specialists who can provide personalized recommendations based on a thorough evaluation, integrating both conventional medical approaches and holistic practices like yoga [7].

## Conclusions

In this study, the utilization of yoga and Yoga Nidra techniques demonstrated a notable enhancement in semen quality in male partners exhibiting abnormal seminal parameters. Although these findings are promising, further inquiry involving larger sample sizes and rigorous controlled trials is warranted to establish a definitive association between these practices and heightened male fertility. Nonetheless, the holistic and noninvasive nature of yoga and Yoga Nidra renders them attractive adjuncts to conventional therapies for male infertility.

## References

[1] Kumar N, Singh AK (2015). Trends of male factor infertility, an important cause of infertility: a review of literature. J Hum Reprod Sci.

[2] Pandi-Perumal SR, Spence DW, Srivastava N (2022). The Origin and Clinical Relevance of Yoga Nidra. Sleep Vigil.

[3] Dhawan V, Kumar M, Deka D, Malhotra N, Dadhwal V, Singh N, Dada R (2018). Meditation & yoga: impact on oxidative DNA damage & dysregulated sperm transcripts in male partners of couples with recurrent pregnancy loss. Indian J Med Res.

[4] Woodyard C (2011). Exploring the therapeutic effects of yoga and its ability to increase quality of life. Int J Yoga.

[5] Darbandi S, Darbandi M, Khorram Khorshid HR, Sadeghi MR (2018). Yoga can improve assisted reproduction technology outcomes in couples with infertility. Altern Ther Health Med.

[6] Ilacqua A, Izzo G, Emerenziani GP, Baldari C, Aversa A (2018). Lifestyle and fertility: the influence of stress and quality of life on male fertility. Reprod Biol Endocrinol.

[7] Sengupta P, Chaudhuri P, Bhattacharya K (2013). Male reproductive health and yoga. Int J Yoga.

[8] Beeder LA, Samplaski MK (2020). Effect of antidepressant medications on semen parameters and male fertility. Int J Urol.

[9] Dada R, Kumar SB, Tolahunase M, Mishra S, Mohanty K, Mukesh T (2022). Yoga and meditation as a therapeutic intervention in oxidative stress and oxidative DNA damage to paternal genome. J Yoga Phys Ther.

[10] Paoletti P, Prediletto R, Carrozzi L (1989). Effects of childhood and adolescence-adulthood respiratory infections in a general population. Eur Respir J.

[11] Muratori M, Marchiani S, Tamburrino L (2015). DNA fragmentation in brighter sperm predicts male fertility independently from age and semen parameters. Fertil Steril.

[12] Cooper TG, Noonan E, von Eckardstein S (2010). World Health Organization reference values for human semen characteristics. Hum Reprod Update.

[13] Muley PP, Gajbe UL, Muley PA (2021). Comparative study of effectiveness of yognidra and antioxidants on semen quality in sub fertile male patients undergoing IVF treatment at Wardha Region, India. JPRI.

[14] Li J, Wu Q, Ng EHY, Mol BWJ, Wu XK, Wang CC (2022). Effects of medicines and supplements on spontaneous pregnancy and semen parameters in male infertility: a systematic review update and network meta-analysis. Engineering.

